# Natural pH-Sensitive Intelligent Edible Gel-Based Packaging: From Structural Design to Fruit Freshness Monitoring

**DOI:** 10.3390/gels12020169

**Published:** 2026-02-14

**Authors:** Tong Zhao, Lulu Wang, Xinyue Wang, Meng Zhang, Xin Zhang, Chen Li, Qian Zhang, Yan Zhao, Lixia Wang

**Affiliations:** 1 Department of Research and Development, Shaanxi Yatai Dairy Co., Ltd., Xianyang 713701, China; 2 College of Life Science, Shaanxi Normal University, Xi’an 710119, China; 3 Shaanxi Engineering Laboratory for Food Green Processing and Safety Control, College of Food Engineering and Nutritional Science, Shaanxi Normal University, Xi’an 710119, China; 4 College of Life Sciences and Food Engineering, Shaanxi Xueqian Normal University, Xi’an 710100, China

**Keywords:** intelligent edible gel-based packaging, natural pH-sensitive indicators, fruit freshness monitoring, sustainable food packaging

## Abstract

The escalating demand for global fruit logistics underscores the urgency of packaging innovations to reconcile preservation efficiency with environmental sustainability, particularly addressing microplastic pollution from conventional plastics and safety hazards posed by synthetic pH-sensitive pigments. Natural pH-sensitive intelligent edible gel-based packaging, which integrates non-toxic indicators into biopolymer gel matrices, offers a viable solution by visually tracking freshness through colorimetric responses to pH fluctuations during storage and transportation. This review systematically synthesizes recent progress in material design, including the development of edible films and coatings, and evaluates the functional mechanisms of natural pH indicators within these systems. Applications across diverse fruit categories demonstrate their efficacy in delaying ripening, inhibiting microbial growth, and signaling quality degradation via dynamic color shifts. Despite enabling real-time, visual freshness monitoring, challenges in mechanical robustness, water resistance, and scalable manufacturing remain. Future advancements should prioritize the integration of multifunctional systems, such as gas conditioning technologies and bioactive components, to enhance practical performance and align with sustainable food preservation objectives, ultimately reducing food waste and elevating consumer safety standards.

## 1. Introduction

The global consumption of fruits has surged in recent decades, driven by increasing awareness of their health benefits, including rich sources of vitamins, dietary fiber, and minerals. However, the perishable nature of fresh fruits poses significant challenges during storage and transportation. The mechanical damage and environmental stress can destroy the natural protective barrier of the fruit peels, accelerating microbial colonization and biochemical spoilage. In the United States alone, approximately 20–30% of post-harvest fruits and vegetables are lost each year due to improper storage during the process from farm to retail [1]. It is noteworthy that improper preservation not only leads to post-harvest losses of fruits and vegetables but also causes severe health and environmental risks due to reliance on non-degradable petroleum-based packaging, which generates toxic substances upon incineration [2].

To address these dual challenges of fruit post-harvest losses and environmental pollution, edible packaging has emerged as a sustainable alternative, which is composed of biodegradable biopolymers (e.g., polysaccharides, proteins, lipids) that form physical barriers regulating gas exchange, reducing water loss, and inhibiting microbial growth [3,4,5]. However, traditional edible packaging can only provide passive preservation and lacks the ability to dynamically monitor the quality changes in fruits during storage. Therefore, the development of pH-responsive intelligent edible gel-based packaging systems (including films and coatings) for active freshness monitoring is particularly crucial [6,7]. Unlike synthetic pH indicators (e.g., Brilliant Yellow), which pose potential toxicity, natural pH-sensitive indicators (e.g., anthocyanins, curcumin) are biodegradable and safer [8,9,10]. These natural pigments can be incorporated into intelligent edible gel-based food packaging to visually indicate freshness levels and acidity levels in fruits and other perishable foods [11].

This review synthesizes recent advances in natural pH-sensitive edible packaging, exploring indicator classification, matrix design, and applications in fruit preservation. Unlike existing studies, it focuses on three aspects: (1) the integration of natural pH-sensitive indicators within edible gel-based matrices (e.g., chitosan, pectin, gelatin) to enhance the intelligent indication feature of packaging; (2) the interaction mechanisms between biopolymer gels and natural pigments, addressing gaps in previous reviews that overlooked matrix-indicator compatibility; (3) the link between material design and industrial scalability, providing a practical method for translating lab-scale prototypes to commercial applications and discusses challenges and future directions, emphasizing the potential of multifunctional, sustainable solutions to reduce food waste and enhance safety. Notably, existing reviews on intelligent edible packaging primarily focus on material characterization rather than practical translation. This review fills this gap by synthesizing material properties and identifying conflicting results in the literature to guide future research. This article aims to offer valuable insights for the technological development and advancement in fruit preservation.

## 2. The Natural Intelligent Edible Gel-Based Packaging

The intelligent edible gel-based packaging is typically constructed using natural biopolymers (e.g., protein, polysaccharide) as the matrices due to their inherent biocompatibility, biodegradability, gelation capability and tunable functional properties [12]. These biopolymers serve dual roles: forming protective barriers against environmental stressors (e.g., oxygen, moisture) and acting as carriers for embedding bioactive indicators in (e.g., pH-sensitive indicators). Typically, these natural biopolymer matrices can be processed into intelligent edible gel-based packaging through methods such as casting, spraying, or extrusion [13]. This forms a selectively permeable layer on the food surfaces, reducing gas exchange between the fruit and the external environment while preventing microbial invasion (Table 1 summarizes the properties essential for natural edible gel-based packaging matrices). Additionally it helps regulate the fruit’s respiration rate to an optimal level, thereby delaying the aging process [14,15].

### 2.1. Polysaccharide-Based Packaging

Natural polysaccharides are often considered as ideal materials for the preparation of edible packaging, as they are safe, stable and possess gel-like properties. A variety of polysaccharides such as starch, chitosan, cellulose and their mixtures are commonly used as substrates for packaging-formation. To enhance the electrostatic attraction and other interactions of polysaccharide-based packages, polar groups within and between molecules are frequently employed. Additionally, plasticizers (e.g., glycerol, sorbitol) [29,30], water-resistant agents (e.g., beeswax, stearic acid) [4,31], and fillers (e.g., nanocellulose) [32,33] can be added in different proportions to adjust the stability and water resistance of these packages. Polysaccharide-based packaging is capable of effectively delaying the adverse changes caused by oxygen and microorganisms in plant products, and it can easily be prepared with good gas resistance for food packaging purposes.

#### 2.1.1. Starch-Based Packaging

Starch, a natural renewable polymer, consists of molecules that are connected through hydrogen bonding interactions between hydroxyl groups. When a starch solution undergoes dextrinization, the intermolecular bonds are broken, and water molecules become bonded to hydrogen bonding sites. This leads to a simultaneous regeneration process of starch molecules. Starch-based packaging is made from dried dextrinized starch solution, making the regeneration process a crucial factor that influences their production. When comparing the film-forming ability of amylose to amylopectin, amylose usually outperforms amylopectin. This is because amylose has the ability to form transparent films with superior mechanical strength and gas barrier properties, whereas amylopectin exhibits inferior performance in these aspects [34]. When starches derived from cassava, wheat, maize, rice, and potatoes are used, they exhibit excellent physicochemical properties such as low pasting temperature and gel stability, which are advantages for creating stable edible films. For example, Veiga-Santos et al. incorporated anthocyanin-rich extracts from grape and spinach into a cassava starch matrix to produce edible films [35]. The resulting intelligent edible gel-based films showcased remarkable antioxidant and pH-sensitive properties, along with enhanced physicochemical properties such as increased flexibility and the formation of uniformly transparent films.

However, the edible packages made of neat starch often exhibit certain limitations in terms of mechanical properties, such as their hydrophilic nature, which hinders their ability to form waterproof food packaging. For instance, cassava starch-based films show higher oxygen barrier properties compared to maize starch films due to their higher amylose content [36]. To overcome hydrophilicity, modification with octenyl succinic anhydride (OSA) reduces water vapor permeability over 52 ± 4% and increases the contact angle (from <40° to 60–80°) [37]. However, OSA modification may reduce the gel-forming capacity of starch, leading to a reduction in anthocyanin entrapment efficiency [38], highlighting the trade-off between hydrophobicity and indicator loading.

Therefore, incorporating a combination of starch with other polymers can be an effective strategy for enhancing the mechanical properties of the films [5]. For example, adding polyvinyl alcohol and other polymers to starch-based films can improve their mechanical strength, waterproofing capabilities, oxygen barrier properties and other desirable characteristics for applications in starch-based packaging [39].

#### 2.1.2. Chitosan-Based Packaging

Chitosan (CS) is a natural cationic polysaccharide extracted from crustaceans. It possesses excellent film-forming, biocompatible, biodegradable, and bioactivity properties. Due to its inherent antibacterial capacity, CS-based packaging presents strong inhibitory effect on bacteria, yeasts and molds [12]. When natural pH-sensitive indicators are incorporated into CS-based food packaging, they allow the indication of physicochemical changes in food products. For instance, Kurek et al. added anthocyanin-rich blueberry and blackberry pomace extracts into CS-based films, and the anthocyanin were continuously released when food was wrapped, allowing the CS films to monitor the freshness of food through their high pH-sensitivity [40]. Chitosan’s performance is highly dependent on its degree of deacetylation (DD): films with DD = 85% exhibit stronger antibacterial activity and higher anthocyanin binding capacity than those with DD = 70%. This is attributed to the higher number of amino groups in high-DD chitosan, which enhance electrostatic interactions with anionic anthocyanins [41]. However, high-DD chitosan films are more brittle, requiring the addition of 5% glycerol as a plasticizer to maintain flexibility without compromising indicator stability [42]. Wan et al. developed CS labels by adding bromocresol blue and methyl red as markers to monitor the concentration of CO_2_ in fruits packaging, thereby reflecting the freshness of the fruits [43]. Wu et al. fabricated multifunctional nanocomposite films by adding *Lactobacillus* peptides and natural plant pigments into a 3D network of pullulan polysaccharide/CS/chitin nanofiber matrix [44]. The films exhibited superior pH-indicator properties, excellent antimicrobial effects, and high-water resistance. Wang et al. synthesized temperature-sensitive visual indicator films by introducing gold nanoparticles into CS-gold nanoparticle composites [45].

#### 2.1.3. Cellulose-Based Edible Packaging

Cellulose, a natural biopolymer composed of β-(1-4)-D-glucopyranose monomers, is highly crystalline and hydrophilic. Due to the inability of cellulose to be directly fabricated into films, it needs to undergo esterification and etherification first in order to enhance its water solubility, flexibility, mechanical properties, and lipid antioxidant power. This process can serve as a preliminary step for the formation of a cellulose-based edible packaging with strengthened mechanical properties [46]. Among the various modified cellulose types, the esterified methyl cellulose and carboxymethyl cellulose (CMC) are the most commonly used cellulose ethers due to their excellent film-forming properties and suitability for direct food contact [47]. Rong et al. utilized a dried mixture of CMC-Na and κ-carrageenan gum to prepare an edible novel label through casting [14]. By incorporating the pH indicator bromothymol blue, the composite CMC-Na film demonstrated excellent intelligent monitoring abilities. Notably, when nanocellulose is decomposed by bacteria, it forms a high-purity film with special multi-vacancy reticulated structures. Embedding or binding indicators into these multi-vacancy reticulated structures can increase the indicator amount and expand the indicator response area, enabling more accurate monitoring of food changes [32,33].

#### 2.1.4. Other Polysaccharide-Based Edible Packaging

In addition to starch, chitosan and cellulose, other polysaccharides such as pectin, seaweed polysaccharide, and plant gums have been utilized as matrices for edible packaging materials. For example, pectin, a carbohydrate polymer abundant in β-(1-4)-linked d-galacturonic acid, has been used to prepare composite films due to its outstanding gel-forming ability [48].

Seaweed polysaccharide, a complex carbohydrate extracted from various types of seaweed or algae, is composed of different monosaccharide units. Seaweed polysaccharide derivatives refer to modified forms of these natural polysaccharides through chemical or enzymatic modifications. The chains of seaweed polysaccharide and their derivatives interact and entangle with each other when dried or cast onto a surface, resulting in the formation of a cohesive thin film upon drying [49]. Additionally, a dense mesh structure will also be constructed when the algal polysaccharide undergoes cross-linking through intermolecular electrostatic interactions, and this structure will promote the mechanical properties of seaweed polysaccharide-based films [50]. In a study conducted by Zhang et al., films were created by combing κ-carrageenan extracted from red algae with anthocyanin molecules [51]. Due to the sulphate-based anionic nature of κ-carrageenan, it readily binds to cationic anthocyanins by electrostatic forces. Therefore, it exhibits a high pH-sensitive capacity, making it suitable for monitoring the freshness of fruits. Additionally, the film also possesses excellent antioxidant properties due to its active ingredients that exhibit a strong capacity for supplying hydrogen. Hydrogen is capable of combining with free radicals and transforming them into more stable products.

As for plant gum matrices, they usually have the ability to form polysaccharide water solutions or polysaccharide derivatives with properties such as safety, film-forming capability, and biodegradability. For instance, gum Arabic exhibits excellent emulsifying, thickening, and stabilizing properties and also shows great potential in edible packaging formation [52]. Coating tomatoes with gum Arabic, as demonstrated by Ali et al., significantly prolonged the quality of the samples and delayed changes in color, weight, hardness, soluble solids concentration, titratable acidity, and ascorbic acid content [53]. Gum Arabic-coated apples and strawberries also showed outstanding effects in preventing decay, which can be attributed to the remarkable antibacterial and antifungal abilities that gum Arabic possesses [54].

### 2.2. Protein-Based Edible Packaging

Most of proteins are easily adhered to the hydrophilic surface of food, providing barriers to O_2_ and CO_2_. However, although protein-based edible packaging exhibits oxygen resistance and transparency, they are unable to prevent water diffusion due to their amphiphilic nature. To address this, denatured proteins caused by high temperature, strong acid, strong base or organic solvents can be adopted to prepare protein-based edible packaging so as to isolate water. The denaturation process stretches the chemical bonds of protein molecules, allowing for the formation of a network structure through intermolecular covalent, ionic and hydrogen bonds, thereby effectively improving water-proof capacity [55]. Usually, animal proteins such as casein, whey protein, gelatin and keratin are commonly used for protein-based edible packaging, while plant proteins such as soy protein, wheat gluten, maize soluble protein and sunflower protein can also be utilized as the basis for protein-based edible packaging materials.

#### 2.2.1. Protein-Based Edible Packaging Derived from Animal Proteins

Animal proteins are primarily obtained from the meat, eggs, and milk of poultry, livestock, fish, and insects. For example, casein molecules—as a main protein composition of milk—contain numerous polar groups, allowing casein-based food packaging to effectively block gases such as O_2_ and CO_2_ from entering or exiting. Whey proteins, as the other main protein in milk, are composed of β-lactoglobulin and α-lactalbumin and possess amphiphilic characteristics with softness and strong oxygen resistance qualities. Novikova and colleagues [56] prepared edible films with a mixture of sodium caseinate and agar, effectively extending the shelf life of food. Marquez et al. utilized transglutaminase-crosslinked whey protein/pectin as a coating agent for fresh-cut fruit and vegetables, resulting in the extended preservation of fresh-cut apples, potatoes, and carrots for up to 10 days [57]. The application of this coating effectively mitigated food weight loss and inhibited microbial growth.

Another natural protein, gelatin, as a partially hydrolyzed protein derived from collagen, exhibits excellent surface activity, thickening properties, and film-forming abilities. As a result, gelatin is commonly employed in the preparation of edible packaging and finds applications in the pharmaceutical industry.

#### 2.2.2. Protein-Based Edible Packaging Derived from Plant Proteins

Among the various plant proteins, bean protein stands out for its excellent emulsifying and film-forming properties. It can be dissolved in acidic or basic solutions to form films by the casting method. Mung bean proteins, for example, have been used by Moghadam and colleagues to prepare anthocyanin-rich edible packaging. The collected films not only exhibited outstanding pH-sensitivity but also demonstrated good water solubility and water vapor permeability [58]. Additionally, the active films showed an improvement in total phenolic content and antibacterial activity.

Zein is a major protein found in maize, which exhibits characteristics of being hydrophobic, thermoplastic, antioxidant, and antibacterial, and also demonstrates excellent film-forming ability with strong waterproof capacity. Pérez et al. improved the properties of tapioca starch films containing *Natamycin* and *Nisin* by adding zein, which enhanced the mechanical properties of the films while reducing stiffness and stress at fracture [59].

Rice protein is a readily available, edible, non-toxic, and biodegradable raw material. It is mainly composed of albumin, globulin, glutelin, and prolamin [60]. The high nutritional value and hypoallergenic nature of the protein make rice protein concentrate an ideal food ingredient. Additionally, compared to its counterparts, rice protein edible film exhibits superior biodegradability. Yan et al. utilized a robust composite film of rice protein hydrolysates and chitosan to enhance the storage quality of fresh salmon [61].

Other plant proteins have also performed well in terms of film-forming capacity. Robles-Flores et al. developed edible coatings with *Cajanus cajan* seed isolate protein and gum, which effectively improved the quality of coated strawberries [62]. Additionally, Kumari and colleagues created novel edible films using fenugreek protein concentrate and tested the physicochemical, mechanical, and thermal properties of the films [63]. The results showed that the fenugreek protein films exhibited good mechanical properties and thermal stability.

### 2.3. Lipid-Based Edible Packaging

Owing to the exhibition of strong hydrophobicity and lipophilicity by the lipid matrix itself, lipid-based edible packaging can be made with a low water vapor transmission rate and high-water penetration resistance, which is highly suitable for fruits to resist water and keep freshness. The most commonly used lipids in packaging include fatty acids, triglycerides, acetylated glycerol monoesters, waxes, and vegetable oils, which are used to preserve fruits and delay their oxidation and deterioration. Formiga et al. coated a mixture of beeswax and hydroxypropyl methylcellulose on the surface of red guava ‘Pedro Sato’, which slowed down weight loss, retained the green color, and enhanced hardness. The coating extended the shelf life of guavas by 6 days [4]. Martinez-Romero et al. coated plums with rosehip oil, which delayed the ripening process and prolonged the quality attributes of plums. On the 14th day, the weight loss rate of the control fruits was 15.6%, while that of the treated fruits was 10.04% [64].

### 2.4. Composite Edible Packaging

While protein-, polysaccharide-, and lipid-based edible packaging each exhibit unique advantages (e.g., oxygen barrier properties of proteins, hydrophobicity of lipids, and gel-forming capacity of polysaccharides), their individual limitations—such as poor water resistance in polysaccharides or brittleness in proteins—have driven researchers to explore composite matrices that synergize the strengths of multiple materials. By combining polymers with complementary physicochemical properties, composite films achieve enhanced mechanical robustness, environmental adaptability, and functional integration, making them ideal carriers for pH-sensitive indicators in fruit preservation. For example, Liu et al. engineered a composite film using beeswax and chitosan via solution casting [65]. Compared to single-component films or blends with gelatin or guar gum, the beeswax–chitosan composite exhibited superior hydrophobicity and mechanical strength, while minimizing water vapor interference with embedded pH indicators. You and colleagues developed a dual-polysaccharide film by integrating konjac glucomannan (KGM) and carboxymethyl cellulose (CMC) as the matrix, with blackcurrant anthocyanins as pH indicators [66]. The KGM-CMC composite film demonstrated excellent thermal stability and pH-sensitive color transitions, while also enhancing the film’s water vapor permeability (from 0.67 to 2.53 g·mm/m^2^ day·kPa), exhibiting inhibitory effects on food-borne pathogens, and stabilizing anthocyanins against thermal degradation.

Similarly, CMC/food-grade agar-based films leverage the high transparency of agar and the mechanical flexibility of CMC, creating a colorimetric display platform with enhanced water resistance and prolonged indicator durability [67]. Beyond this, composite matrices like sodium alginate (NaAlg) and sodium carboxymethyl cellulose (NaCMC) hydrogel beads, originally designed for controlled drug release [68], highlighted the potential of polysaccharide composites to encapsulate pH-sensitive pigments while enabling tunable release kinetics, a feature adaptable to fruit coatings requiring sustained antimicrobial action.

### 2.5. Interaction Mechanisms Between Biopolymer Gel Matrices and Natural pH Indicators

The functionality and reliability of natural pH-sensitive indicators in intelligent edible gel-based packaging are largely determined by their interaction with biopolymer gel matrices. These interactions not only affect the dispersion uniformity of indicators but also regulate their stability, migration behavior, and long-term color response accuracy. Typically, three main modes dominate the key interaction pathways.

(1) Electrostatic Interactions

Cationic biopolymers (e.g., chitosan) form complexes with anionic natural indicators (e.g., anthocyanins from red cabbage) via electrostatic attraction. This interaction reduces the mobility of indicator molecules, preventing their migration to fruit surfaces and enhancing color stability under light exposure. For example, chitosan’s amino groups (+) bind to anthocyanins’ carboxyl groups (−), forming a stable gel network that traps indicator molecules [69].

(2) Hydrogen Bonding

Polysaccharides (e.g., starch, carboxymethyl cellulose) and proteins (e.g., gelatin) form hydrogen bonds with hydroxyl (-OH) or carbonyl (C=O) groups of indicators (e.g., curcumin, betanin). This bonding improves the water resistance of the gel matrix and slows indicator degradation [70,71,72]. For example, starch’s hydroxyl groups form hydrogen bonds with curcumin’s phenolic hydroxyl groups, reducing curcumin’s sensitivity to moisture [70].

(3) Gel Network Entrapment

Usually, the biopolymer gel matrices entrap indicator particles by forming a 3D porous structure physically [72]. The pore size of the gel network is critical: too small may hinder pH-responsive color changes, while too large leads to rapid indicator leakage and reduced long-term reliability.

Additionally, since fruit storage and transportation often involve temperature and humidity fluctuations, these conditions may disrupt matrix-indicator interactions [73]. Rapid temperature changes damage the gel’s 3D network structure, causing indicator leakage, while high humidity weakens bonding, leading to color fading. To address this, Kazemi et al. enhanced network stability by adding beeswaxes to reduce water absorption [31].

## 3. Natural pH-Sensitive Colorant Indicators for Intelligent Edible Gel-Based Packaging

Natural pH-sensitive indicators are the core components of intelligent edible gel-based packaging. They visual track fruit freshness by changing color in responses to pH fluctuations during storage. These indicators are taxonomically grouped by their chemical structures, which dictate their chromatic behavior and compatibility with edible matrices.

Based on their chemical structures, natural pH-sensitive colorant indicators can be categorized into various groups. (1) Conjugated systems, such as anthocyanins (E 163), betanin (E 162), and carotenoids (E 160a), dominate due to their extended π-electron delocalization, allowing pronounced color shifts across pH gradients; (2) metal-coordinated porphyrins like chlorophylls (E 140) leverage central metal ions (e.g., magnesium in chlorophyll) to modulate chromophore stability under acidic or alkaline conditions; (3) diverse derivatives-phenolic compounds, curcumin, and others, whose functional groups (e.g., hydroxyl, ketone) undergo protonation/deprotonation reactions to trigger color changes [10,74].

Among these, anthocyanins, betanin, and curcumin stand out for their robust pH-sensitivity, natural abundance, and additional bioactivities (e.g., antioxidant, antimicrobial properties), making them ideal for translating into packaging applications [74].

### 3.1. Anthocyanin

**Figure 1:**
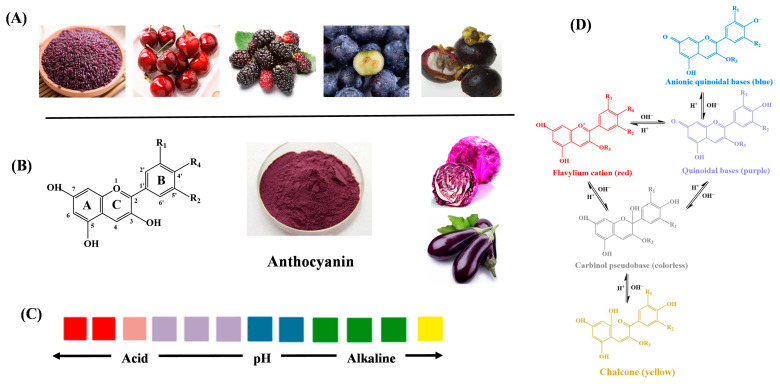
The sources (**A**), structure (**B**) of anthocyanin, color changes (**C**), and structural transitions (**D**) under reactions from acid to alkaline [77].

Anthocyanins are natural flavonoids found in fruits, vegetables, and flowers (Figure 1A). They are water soluble and have vibrant colors. Structurally, most anthocyanins are 3-glucosides of anthocyanidins (sugar-free aglycone), with a core C6-C3-C6 carbon skeleton consisting of an A ring (C6), a C ring (C3), and a B ring (C6) (Figure 1B). Substitutions at the B ring (at the R1 and R2 positions), such as methylation or hydroxylation, generate structural diversity: over 650 anthocyanin variants have been identified in nature, with six common types including geranium pigment, centaurea pigment, delphinium pigment, peony pigment, morning glory pigment, and mallow pigment [75].

Anthocyanins derive their pH-sensitive chromaticity from a dynamic equilibrium between multiple structural forms, governed by their core C6-C3-C6 flavonoid skeleton (Figure 1B). The B ring (R1 and R2 substituents) plays a critical role in modulating pH sensitivity: hydroxyl groups (-OH) at positions 3′, 4′, or 5′ enhance acidity through hydrogen bonding, while methoxyl groups (-OCH_3_) at these sites increase stability in neutral-to-alkaline environments [76].

This core C6-C3-C6 flavonoid skeleton, with structural plasticity, enables anthocyanins to exhibit pronounced color transitions across a broad pH range, from dark red to dark blue to yellow, across a pH range of 1–14 (Figure 1C) [76]. This is attributed to the high sensitivity of anthocyanins towards pH variations. In strongly acidic conditions (pH < 3), anthocyanin exist predominantly as the “flavylium cation”, featuring a planar structure that absorbs blue light, yielding vivid red colors. As OH^−^ concentration increases (pH 4–6), deprotonation at the C-ring hydroxyl group induces a non-planar quinoidal structure “quinoidal base”, which absorbs green light and appears purple to blue. At neutral pH (pH 7–8), hydration at the C2 position forms the “carbinol pseudobase”, a colorless, non-chromophoric structure that causes transient color fading. With further elevation of OH^−^ concentration, in alkaline environments (pH > 8), ring opening generates the “chalcone forma”, a linear chalcone structure with extended conjugation that absorbs yellow light, manifesting as pale yellow or brown (Figure 1D) [75,77].

This pH-sensitive characteristic makes them suitable for rapid monitoring of food freshness [10,77]. For example, Yoshida and colleagues developed intelligent natural depolymerized chitosan films incorporating anthocyanins as acid-base indicators, which exhibited vivid color transitions, pink in acidic conditions, teal in neutral environments, and yellow in alkaline states [78]. These responses directly reflect pH changes during fruit spoilage, enabling visual quality assessment.

In addition, anthocyanins possess significant antioxidant activity, including anti-inflammatory, anti-tumor, antibacterial, and neuroprotective effects, making anthocyanin-added packaging possess excellent bioactivities. Moghadam et al. demonstrated that anthocyanin-added films showed significant antibacterial activity, particularly against *Escherichia coli* and *Staphylococcus aureus* [58]. However, it should be noted that the acid-base indicator properties of anthocyanins are influenced by other factors: (1) The extracted source plays a significant role. For example, anthocyanins from red cabbage (rich in cyanidin-3-glucoside) show a broader pH response range (pH 1–12) and higher color stability than those from strawberries (rich in pelargonidin-3-glucoside) [77]. This difference arises from the number of hydroxyl groups on the B-ring: cyanidin-3-glucoside has three hydroxyl groups, enhancing hydrogen bonding with gel matrices, while pelargonidin-3-glucoside has only two [79]. In practical applications, red cabbage anthocyanins are preferred for long-term storage (≥15 days), while strawberry anthocyanins are suitable for short-shelf-life fruits due to their faster color response [80]. Anthocyanins extracted from red cabbage display red at pH 1–2, pink at pH 3, purple at pH 4–6, blue at pH 7–8, green at pH 9–11, and yellow at pH 12 [77]. Anthocyanins extracted from carrots exhibit an orange red to yellow color across the pH range of 2–12 [81]; (2) the color stability of anthocyanins in food is closely linked to storage temperature, for instance, the color stability test showed that the films incorporated with anthocyanins were stable at refrigeration temperature and room temperature for up to 14 days, with relative color changes below 5% [82]; (3) due to the hydrophilic nature of anthocyanins, excessive addition compromises the water vapor barrier performance of packaging films [83].

### 3.2. Betanin

**Figure 2:**
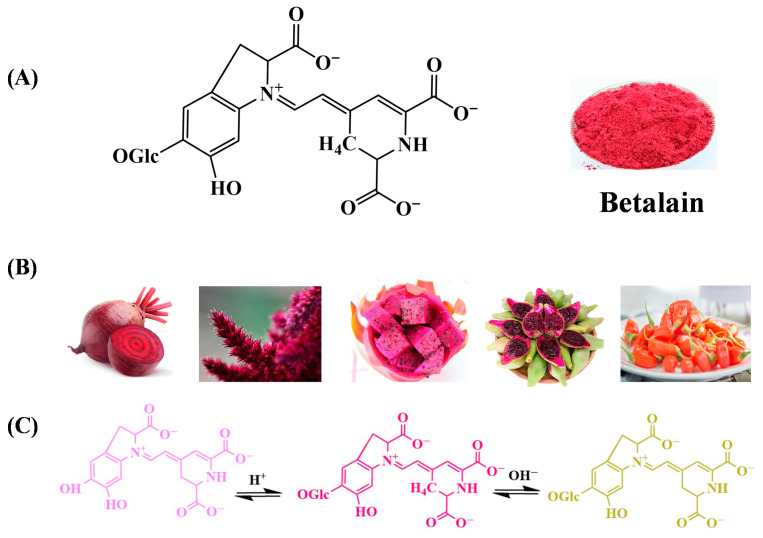
The structure (**A**), source (**B**) of betanin, and structure transitions under reactions from acid to alkaline (**C**) [94].

Betanin is a water-soluble nitrogenous compound with a red-purple hue that is widely present in nature. It is predominantly found in beets (the primary source of betanin) [84], as well as dragon fruit, pear cactus, and amniotic fluid (Figure 2A,B). Betanin belongs to the betacyanins class of betalains, which are characterized by their red-purple color, while betaxanthins (yellow color) constitute another class within the betalains family. Structurally, betalains are derivatives of betalamic acid [4-(2-oxoethylidene)-1,2,3,4-tetrahydropyridine-2,6-dicarboxylic acid] conjugated with amino acids or amines [85]. Typically, betanin presents a red-purple hue due to the conjugation of betalamic acid with *cyclo*-dopa-5-O-glucoside. The presence of various chromophores in the structure of betalain, such as C=C, C=N, and C=O groups, imparts excellent UV protection properties. Interestingly, the color and structure of betanin are influenced by environmental pH levels [86].

Typically, betanin displays violet hues under strongly acidic conditions (pH < 3), transitions to red-purple in the neutral range (pH 3.5–7), and degrades to yellow-brown in alkaline environments (pH > 7) (Figure 2C). These excellent pH-sensitive color transitions of betalains (including betanin) are rooted in its unique molecular architecture and electronic effects [84].

At low pH (<pH 3), the immonium group in betanin undergoes protonation, forming a cationic species where the positive charge delocalizes across the conjugated system of betalamic acid and *cyclo*-dopa-5-O-glucoside. This cationic form exhibits a red-to-violet hue due to enhanced π-π stacking and extended conjugation, which shifts the light absorption maximum to shorter wavelengths (480–550 nm) [87]. As pH increases to neutral ranges, the molecule exists as a zwitterion, stabilizing the red-purple chromophore through intramolecular hydrogen bonding between the carboxyl group of betalamic acid and the hydroxyl group of *cyclo*-dopa-5-O-glucoside. At high pH (>pH 7), the aldimine bond (C=N) in betanin undergoes nucleophilic attack by hydroxide ions, triggering hydrolytic cleavage that dissociates betalamic acid from *cyclo*-dopa-5-O-glucoside (Figure 2C). This bond rupture disrupts the conjugated system, converting the planar chromophore into a non-planar structure with reduced π-electron delocalization, resulting in a yellow-brown color due to increased absorption in the 380–450 nm range [77,78]. The optimal stability of betanin in pH 3.5–7 is attributed to the balance between protonation states and hydrogen bonding, which safeguards the integrity of the conjugated immonium chromophore [77,88].

The stability of betanin in the pH 3.5–7 range is attributed to the equilibrium between the planar conjugated immonium structure and intramolecular hydrogen bonding, which protects the aldimine bond from premature cleavage [89]. However, like most natural pigments, betanins are susceptible to oxygen, temperature, and light, which disrupt their chromophore integrity and induce color degradation. Temperature is a critical stability factor: at pH 7, betanin is stable for ≥20 days at 4 °C and >275 days at −30 °C, but degrades rapidly above 50 °C [90]. For example, boiling red beet for 180 s reduces betacyanin content by 51%, and 30 min treatment at 125 °C leads to 81% betacyanin loss [91]. Oxygen also accelerates degradation (mediated by reactive oxygen species [ROS]) [92], but stability improves under nitrogen atmospheres or low-oxygen storage [90]. Notably, betalain degradation products may retain or enhance antioxidant activity [93], a unique advantage for packaging applications. When exposed to oxygen, oxidative degradation is initiated, reactive oxygen species (ROS) attack the double bonds in the betanin acid moiety, breaking the conjugated system and leading to the formation of colorless byproducts. Temperature elevation accelerates this process by increasing molecular vibrations, weakening hydrogen bonds and promoting aldehyde bond hydrolysis. Light—particularly ultraviolet (UV) radiation—induces photodegradation by exciting electrons in the chromophore, causing bond isomerization and irreversible structural damage. These factors can disrupt their chromophore structure and result in noticeable color changes from red to a dull light brown. Therefore, it is important to consider the application conditions for betanin [94].

As for biological functionality, betanin possesses antioxidant and antibacterial properties, making it suitable for incorporation into thin films to provide bioactivity [95]. Ghasempour et al. incorporated betanin liposomes into a composite packaging film comprising whey protein isolate and Persian gum [96]. The film exhibited antibacterial activity against *Staphylococcus aureus*, along with enhanced antioxidant activity. Furthermore, the film extended the shelf life of perishable food products and provided pH indication for such food products. Another study introduced betanin to a gelatin/chitosan nanofiber/ZnO nanoparticles bionanocomposite film to investigate its impact on fresh food preservation [97]. The findings demonstrated that the fabricated film possessed remarkable water resistance and displayed discernible color variations at different pH levels.

### 3.3. Carotenoids

Carotenoids are a class of tetraterpenoid organic pigments primarily localized in the chromoplasts of plants and some photosynthetic algae, bacteria, and fungi [98]. Structurally, carotenoid molecules consist of a polyene backbone with four terpenoid moieties, each comprising 10 carbon atoms [99]. The terminal moiety can be cyclic, resulting in a total of 40 carbon atoms within the molecule. Carotenoids can be categorized into two groups based on their oxygen content: non-oxygenated carotenes (e.g., lycopene and β-carotene) and oxygenated xanthophylls (e.g., lutein, zeaxanthin and astaxanthin) [50].

Carotenoids typically exhibit vibrant red, orange, and yellow hues. Due to the presence of multiple unsaturated double bonds in the main chain of carotenoid molecules, they display high susceptibility to oxygen, temperature fluctuations, and light exposure. In acidic environments, protonation of oxygenated xanthophylls (e.g., lutein) occurs at hydroxyl or epoxy groups, disrupting the electron delocalization along the conjugated double bonds. This leads to cis–trans isomerization specifically, the conversion of stable all-trans configurations to cis isomers, which shortens the effective conjugation length and shifts light absorption to shorter wavelengths, causing color fading or hue shifts (e.g., from deep red to orange) [100,101].

Under alkaline conditions, deprotonation and saponification of esterified carotenoids (e.g., astaxanthin esters) trigger de-esterification, generating free acids that aggregate and increase solution turbidity [102]. Thus, the loss of turbidity can serve as an indicator for assessing carotenoid behavior under various pH conditions. Concurrently, hydroxide ions attack the double bonds, inducing oxidative cleavage of the polyene chain and loss of chromophore integrity, resulting in rapid color degradation [75]. These mechanisms collectively explain why carotenoids exhibit optimal stability at neutral pH (pH 7) but degrade drastically under acidic (pH < 5) or alkaline (pH > 8) conditions.

The pH-dependent color changes in carotenoids make them suitable for developing visual quality-monitoring tools. Medina-Jaramillo reported that extracts from green tea and basil, rich in carotenoids, exhibit distinct color transitions with pH variations, highlighting their potential in pH-sensitive colorimetric indicator films [103]. Such systems can visually signal food spoilage by reflecting changes in environmental pH, offering a natural and cost-effective solution for real-time freshness assessment.

### 3.4. Chlorophyll

**Figure 3:**
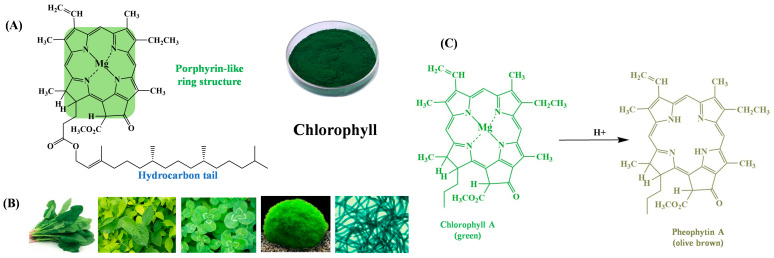
The structure (**A**), source (**B**) of chlorophyll, and structural transitions in acid (**C**) [109,110].

Chlorophyll is a hydrophobic green pigment present in higher plants and all other photosynthesizing organisms. It exhibits insolubility in water and possesses various physiological activities, including antioxidant, anti-inflammatory, and antimutagenic effects [104]. In terms of structural composition, chlorophyll comprises a fundamental unit consisting of a porphyrin ring and an elongated hydrocarbon side chain known as phytol. Despite the existence of various types such as chlorophyll a, b, c, and d, along with bacterial chlorophyll and green algae-derived chlorophyll, their structural differences are minimal. All variants consist of a porphyrin ring ‘head’ and a phytol ‘tail’ (Figure 3A) [105].

The pH-dependent color transitions of chlorophyll are rooted in structural modifications of its porphyrin ring and phytol tail. In acidic environments, hydrogen ions (H^+^) attack the central magnesium (Mg^2+^) ion in the porphyrin ring, triggering a ligand exchange reaction where Mg^2+^ is replaced by two H^+^ ions, forming pheophytin (Figure 3C). This substitution disrupts the planar geometry of the porphyrin ring, reducing π-electron delocalization and shifting light absorption from the characteristic green band (660–670 nm) to a broader, less intense spectrum, resulting in olive green or brownish-green hues [106,107].

Conversely, under alkaline conditions (pH > 7), the ester bonds between the porphyrin ring and phytol tail undergo saponification, hydrolyzing into water-soluble chlorophyllin salts (e.g., sodium or potassium chlorophyllin). This reaction removes the hydrophobic phytol tail, increasing solubility in aqueous environments while preserving the conjugated porphyrin structure, thus maintaining a vibrant green color (Figure 3) [108]. The stability of chlorophyll in alkaline solutions is attributed to the negatively charged carboxylate groups formed during saponification, which repel hydroxide ions and protect the porphyrin ring from further degradation.

Notably, chlorophyll is inherently unstable and susceptible to degradation when exposed to light, heat, oxygen, and oxidizing agents. Light-induced photodegradation occurs through singlet oxygen formation, which damages the porphyrin ring’s conjugated system and accelerates Mg^2+^ loss. Elevated temperatures exacerbate this process by enhancing molecular vibrations, weakening ester bonds in the phytol tail and promoting saponification even under neutral pH conditions. Oxygen and oxidizing agents further compromise chlorophyll integrity by initiating radical-mediated attacks on double bonds in the porphyrin ring, leading to pigment bleaching and loss of functional activity [106,107].

### 3.5. Curcumin

Curcumin is a phytochemical phenolic extracted from turmeric with three unstable hydrogen atoms, meanwhile it is a symmetric molecule with two methoxyphenyl groups connected by a seven-carbon chain and an enol form of β-diketone [111]. The phenolic hydroxyl groups of curcumin can react with environmental hydroxide ions to form phenoxide anions, endowing curcumin with high sensitivity and color changes across different pH levels. This chemical reactivity arises from the dynamic balance between its enol and ketone tautomeric forms, making it a versatile pH indicator [112,113].

**Figure 4:**
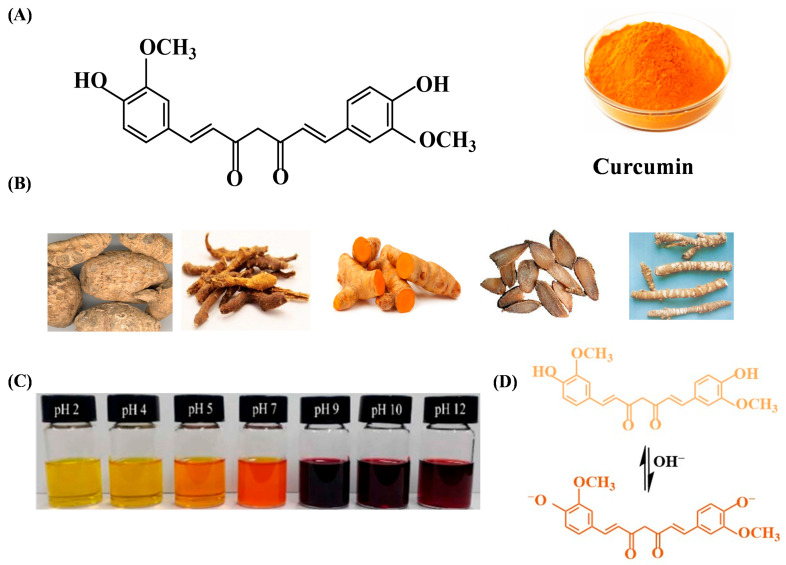
The structure (**A**), source (**B**) of curcumin, the color changes (**C**) and structure transitions (**D**) in alkaline [116].

At low pH (1–7), curcumin exists predominantly in the enol form, stabilized by intramolecular hydrogen bonding between the β-diketone oxygen and the phenolic hydroxyl group. This planar structure exhibits a yellow hue due to strong absorption in the 420–450 nm range, driven by π-π transitions in the conjugated seven-carbon chain linking the two methoxyphenyl rings [113]. As pH increases above 8.5, the phenolic hydroxyl groups (-OH) lose protons, forming a phenoxide anion that triggers tautomerization to the ketone form. This structural shift extends the conjugated system, allowing greater electron delocalization across the entire molecule and shifting light absorption to longer wavelengths (500–550 nm), resulting in a vivid crimson color (Figure 4C,D) [113]. The reversible nature of this transformation-mediated by hydrogen ion concentration enables curcumin to act as a sensitive pH indicator, with color changes directly reflecting environmental alkalinity [112].

The pH-sensitive color transitions of curcumin have been leveraged in diverse edible packaging systems, including curcumin/polysaccharide, curcumin/protein, and curcumin/protein-polysaccharide composite films. These packaging materials take advantage of curcumin’s ability to undergo visible color changes in response to pH fluctuations during fruit spoilage, providing real-time freshness monitoring [112,113]. For example, Ghosh et al. incorporated curcumin-doped functionalized cellulose nanofibers into edible chitosan coating for kiwifruit [114]. The addition of curcumin has a yellowing effect on edible coating materials, and the coated test fruit is enabled to present color changes during storage, so as to signal microbial-induced pH shifts. In addition, a smart packaging system with curcumin as an indicator incorporated into the film can also serve as an effective indicator for monitoring the freshness of most animal-based protein foods, such as fish, pork and shrimp [115].

### 3.6. Alizarin

Alizarin (1,2-dihydroxyanthraquinone, C_14_H_8_O_4_) is an orange crystalline pigment that is typically found naturally in the roots of madder plants. Alizarin’s pH sensitive color transitions are governed by the deprotonation of its phenolic hydroxyl groups, which enhances molecular conjugation through resonance between the resulting oxyanions and adjacent carbonyl groups. Under alkaline conditions, the ionization of hydroxyl groups extends the π-conjugated system, shifting the absorption maximum to longer wavelengths and causing a chromatic transition from yellow to purple.

**Figure 5:**
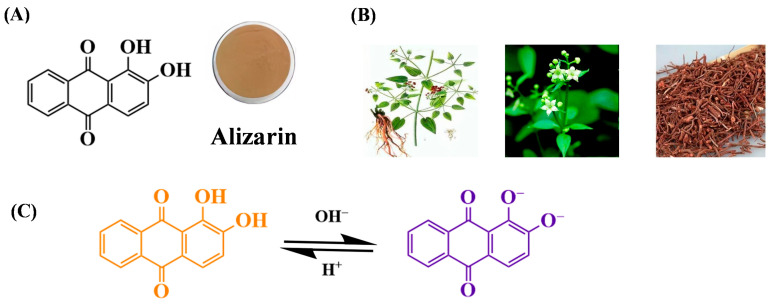
The structure (**A**), source of alizarin (**B**) and structure transitions under reactions from acid to alkaline (**C**) [117].

As pH increases (pH < 7), the first phenolic hydroxyl group dissociates, releasing a proton and forming a monoanion. The negative charge delocalizes across the anthraquinone ring via resonance, extending the conjugated system and shifting light absorption to 500–550 nm, which manifests as an orange hue. As pH value increases from 6 to 9, the first phenolic hydroxyl group of alizarin dissociates to form a monoanion. The negative charge delocalizes across the anthraquinone ring via resonance, extending the π-conjugated system and shifting the absorption maximum to 430–480 nm, which corresponds to a purple color (Figure 5). At pH > 10, the second hydroxyl group dissociates, generating a dianion with full π-electron delocalization over the entire molecule. This planar configuration further red-shifts the absorption to 500–520 nm, resulting in a deep red hue due to reduced energy gaps in π-π transitions [65,117]. The stepwise deprotonation and conjugation expansion thus drive the chromatic transition from yellow (neutral) to purple (monoanion) and finally to red (dianion).

Beyond its pH-sensitive coloration, alizarin offers additional functional benefits derived from its molecular structure “the anthraquinone ring” confers anti-ultraviolet (UV) properties by absorbing UV radiation (200–400 nm), protecting packaged foods from photooxidation [118].

The combination of pH-sensitive color changes and intrinsic functionalities has spurred the development of alizarin-based packaging materials. For example, Liu et al. developed an alizarin indicator using starch cellulose paper and cellulose paper as the foundation—through the coating method—to serve as a pH-sensitive indicator for intelligent edible gel-based packaging systems [116]. Wu and colleagues designed a poly (lactic acid)-based functional partition composite membrane containing chitosan and alizarin using the solution casting method [119]. When the environmental pH changed repeatedly, the membrane showed reversible color change to yellow under acidic condition and purple under alkaline condition.

### 3.7. Shikonin

**Figure 6:**
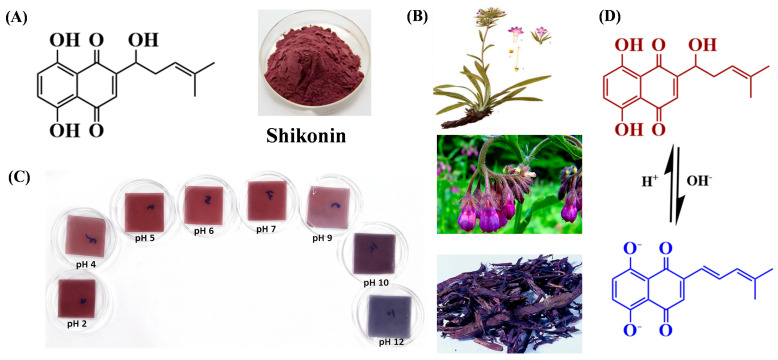
The structure (**A**), source (**B**) of shikonin, color changes (**C**) and structure transitions (**D**) under reactions from acid to alkaline [126,127].

Shikonin, a lipophilic naphthoquinone compound extracted from the root of *Arnebia euchroma*, exhibits diverse bioactivities including antibacterial, anti-inflammatory, wound healing, and anticancer effects [120]. Its main chain consists of alternating 1,3-linked-D-galactose and 1,4-linked 3,6-anhydro-L-galactose units, with a complex multiphase structure [116]. Like other natural pH-sensitive colorants, shikonin has also attracted research interest in intelligent edible gel-based packaging due to its acid-base indicator properties [121]. The color changes and structure transitions of shikonin under reactions from acid to alkaline are shown in Figure 6C,D.

Shikonin’s pH-sensitive color transitions are governed by the protonation state of its naphthoquinone ring and hydroxyl groups (Figure 6D). In acidic environments (pH < 5), the β-hydroxyl group and the keto group in the naphthoquinone moiety undergo protonation, forming a stable enol structure that enhances π-electron delocalization across the ring system. This configuration absorbs light in the 480–520 nm range, yielding a deep red hue due to intensified charge-transfer transitions [122]. As pH increases to alkaline conditions (pH > 7), the hydroxyl group dissociates, releasing a proton and generating a phenoxide anion that triggers tautomerization to the ketone form. This structural shift reduces π-conjugation and increases the energy gap for electron transitions, shifting light absorption to 550–600 nm and manifesting as a purple-blue color [121,122].

The reversible enol-keto tautomerization, driven by hydrogen ion concentration, allows shikonin to act as a sensitive pH indicator, with color changes directly reflecting environmental alkalinity [121,123,124]. For example, Zou et al. prepared a dual-functional nanofibrous film loaded with shikonin in quaternized chitosan/polycaprolactone, which demonstrated pH-sensitivity for active and intelligent edible gel-based food packaging [125].

Although intelligent edible gel-based packaging containing natural pH indicators can visualize fruit freshness and exert varying degrees of bioactivity, it is important to note that different combination methods of these natural pH indicators with edible packaging have their own advantages and disadvantages. We categorize the mainstream combination methods into three types: simple mixing, covalent binding, and encapsulation, as summarized in Table 2.

**Table 2:** Combination methods of intelligent edible gel-based packaging with natural pH indicators.

Combination Method	Operation Mode	Advantages	Disadvantages	References
Simple mixing	Directly mix and stir uniformly to form films/coatings	Easy to operate, low cost, suitable for preliminary large-scale application	Uneven dispersion and easy to agglomerate; poor long-term stability	[106,128]
Covalent binding	Combined by cross-linkers and forming covalent bonds between indicator pigment functional groups and biopolymer base	Excellent stability and high reliability of color response	Complex operation requiring purified natural indicator pigments; potential residual cross-linkers; possible bioactivity loss post-binding	[106,116]
Gel encapsulation	Encapsulate into biopolymer gel-based networks and then composite into packages	Illustrate physical protection; controlled release; no migration risk	Tedious preparation process; high cost	[70,129]

## 4. Preparation Methods of Intelligent Edible Gel-Based Packaging

Intelligent edible gel-based packaging comprises “coatings” and “films” two distinct categories. Coatings are thin layers directly deposited on food surfaces (e.g., via spraying, dipping, or fluidized-bed coating), while films are freestanding structures fabricated by molding (e.g., casting, extrusion, or electrospinning). The two differ in preparation strategies and functional characteristics.

Three common fabrication techniques for intelligent edible gel-based packaging are summarized below: (1) Casting and continuous casting (solution-based film formation); (2) extrusion (melt-processing for industrial scalability); (3) spraying and electrostatic spraying (surface-coating to enhance functionality). Each method is analyzed for its technical principles, and features in intelligent edible gel-based food packaging.

### 4.1. Casting and Continuous Casting

Casting is a simple but cost-effective method for preparing films; it typically refers to the process of pouring liquid or slurry materials into a mold or onto a flat surface (such as glass plates) to solidify and form (12–24 h) [14,130]. The process of casting mainly consists of four steps. Firstly, the raw materials are continuously stirred or subjected to ultrasonic treatment to obtain a homogenized and stable solution. Secondly, the film solution is centrifuged and poured onto glass plates, allowing it to evaporate and dry in desiccators. Subsequently, the dried films need to be peeled off and neutralized with sodium hydroxide to remove excess acid. Finally, films need to be cleaned and placed in a desiccator, maintaining them in a constant temperature and humidity chamber. The casting method offers a convenient way to produce films with desired properties [131]. It allows for precise control over the film thickness and facilitates the incorporation of various additives or active compounds, but requires careful drying and storage to maintain film stability and quality [132]. Rong et al. adopted CMC-sodium and carrageenan to prepare edible films by the casting method, incorporating bromothymol blue as a pH-sensitive indicator to enable the detection of freshness in freshly cut fruits [14].

Despite its advantage of low-temperature production, casting suffers from inherent limitations: lengthy drying period, a discontinuous operational mode, and poor scalability for industrial application [132]. To address these challenges, the continuous casting method has been developed as an optimized alternative, employing an automated, uninterrupted production line to achieve large-scale manufacturing. By utilizing a conveyor belt to deliver film solution to a drying chamber, it overcomes these shortcomings and results in the rapid collection of dried films (1–3 h) [131]. Compared to conventional casting, continuous casting eliminates the need for manual handling of individual substrates and ensures uniform film thickness through precise control of conveyor speed and drying temperature [133]. However, although continuous casting has been used in edible film producing for decades, its application in fruit freshness preservation and quality monitoring remains limited, which needs to be explored further in the future [133]. Industrial-scale continuous casting systems feature automated, uninterrupted production, offering higher efficiency (rate of 30–40 m^2^/h) and more uniform film thickness (±0.03 mm) compared to traditional batch casting (±0.06 mm) [133,134]. Key operational parameters include conveyor belt speed (1.2–1.8 m/min) and drying temperature (30–40 °C) for gel-based matrices, excessive drying temperatures can disrupt the gel network structure, leading to reduced indicator stability [135,136]. From an industrial perspective, continuous casting reduces production costs (20–40%) compared to batch casting due to lower labor and energy consumption. However, the need for specialized equipment and upfront investment constitutes a barrier for small-to-medium enterprises, particularly those focusing on fruit preservation applications [137].

### 4.2. Extrusion Method

Extrusion method is a dry process that utilizes an extruder to produce films. Owing to the minimal requirements for film solution, the film solution can be evaporated and dried rapidly through the extruder. The extruder consists of a feeding zone, mixing zone, and heating zone, with a film-forming extrusion tool located at the end of the extruder to regulate the shape and thickness of films. In comparison to casting, the extrusion method offers faster production and greater energy efficiency in film making. However, it is not suitable for heat-sensitive materials since the high-temperature treatments involved in the extrusion procedure will denature those materials and hinder film formation [138]. Ceballos et al. fabricated hydrolyzed starch-based pH-sensitive films through extrusion method and press molding with anthocyanins as pH indicators [139]. Cheng et al. incorporated three natural waxes (beeswax, candelilla wax, and carnauba wax) into starch/gelatin film solutions and obtained edible films with highly hydrophobic surfaces by using the extrusion and blowing method [15]. It is worth noting that during the extrusion process, careful attention must be given to adjusting relevant parameters in a timely manner, because the extrusion temperature, pressure, extrusion rate, and pulling rate are extremely critical factors in film making [138].

### 4.3. Spraying and Electrostatic Spraying

Spraying is the most popular method used to coat fruits, due to its ability to produce uniform and consistent thin films. The spraying system typically consists of high-pressure spray applicators and air-atomizing systems, which can atomize liquid droplets with strong force through a specialized nozzle [140]. During the spraying process, a high-pressure spraying gun generates fine atomized droplets of the coating solution. Then, the atomized droplets spread evenly on the surface of fruits and form a thin layer after drying [141].

Despite the deposition of a thin layer on the fruit surface during the spraying method, there is still a considerable amount of atomized liquid that fails to reach the target area, resulting in material wastage. To address this issue, an optimized technique known as electrostatic spraying has been developed as a modification of the spraying method. Electrostatic spraying offers higher transfer efficiency and a more uniform distribution of the spraying material. In electrostatic spraying, an intense electrical field needs to be applied to the coating material, so as to force it away from the electrode. And the charged coating material needs to carry an opposite charge to the nearest grounded target area, which allows these charged droplets to be attracted and dispersed on the surface of the target food via electrostatic attraction. Peretto et al. utilized the electrostatic spraying method to preserve fresh strawberries by coating their surfaces with sodium alginate [142]. The study found that electrostatic spray applications exhibit a significant improvement in transfer efficiency compared to non-electrostatic spraying coatings. Meanwhile, droplet deposition on the target surface was increased with a more even distribution. This led to a remarkable extension of the strawberries’ shelf life. Owing to its quick and relatively simple application, electrostatic spraying is highly suitable for coating fresh products during preservation. Furthermore, advancements in electrostatic spraying have expanded its applicability beyond liquid coatings, now allowing for the effective application of powder coatings as well [3].

In addition to the casting, extrusion, and spraying techniques discussed earlier, several other molding methods have been reported for edible gel film fabrication (e.g., 3D printing and compression molding). Table 3 compares these molding techniques for edible films/coatings with synthetic film preparation methods.

## 5. Applications of Intelligent Edible Gel-Based Packaging Films Containing Natural pH Indicators in Fruit Preservation

Fruits are prone to spoilage caused by microorganisms, enzymes, physiological decay, and exposure to oxygen during storage. To extend their shelf lives and assess their freshness, maturity level, and overall quality, pH-sensitive intelligent edible gel-based packaging based on natural pigments can be employed. Due to the variety of fruits, they were classified into two categories: fresh whole fruits and fresh-cut fruits (as shown in Table 4), and a summary is provided of the applications of the intelligent edible gel-based packaging films containing natural pH indicators in fruit preservation. Interestingly, the polysaccharide-based packaging is still mostly used in freshness keeping and indicators monitoring fresh fruits.

In berries, Haque et al. used purple cabbage, which is a rich source of anthocyanin, and garlic oil with antibacterial properties to prepare starch-based films [80]. The films they made were able to detect the spoilage in strawberries through color change, transitioning from purple to pink-purple. Notably, the effectiveness of pH-sensitive packaging varies by fruit respiration intensity: for high-respiration fruits (e.g., strawberries, O_2_ consumption rate 20–40 mL/kg·h at 25 °C), chitosan-based films with purple cabbage anthocyanins extend shelf life by six days (from 3 to 10 days) [143,144]. For low-respiration fruits (e.g., grapes, O_2_ consumption rate 10–15 mL/kg·h at 25 °C), carboxymethyl cellulose (CMC) films with pomegranate anthocyanins perform better, prolonging shelf life by 25 days (from 15 to 40 days) due to their higher gas barrier properties at 0–5 °C [66]. Fatma et al. added pomegranate anthocyanin extract to edible and flexible films composed of carboxymethyl cellulose for packing red grapes and plums [145]. The results illustrated that the fabricated films exhibited rapid responsiveness to pH changes, with color changes from apple-red (pH 2) to moss green (pH 12) occurring within a few seconds. At pH 2, the apple-red color film appears. When the pH rises, the color turns light pink. At pH levels between 6 and 8, the film turns caramel. At pH 10, the color changes to light brown, and at pH 12, it becomes moss green.

In melons, Netramai et al. developed a colorimetric polysaccharide-based film (contained carboxymethyl cellulose, carrageenan, and pectin) with butterfly pea (*Clitoria ternatea* L.) flower extract to protect and indicate the quality changes in honey dew melon [146]. The films they made illustrated rapid pH-sensitivity, with their color changing from magenta to purple to blue as the pH increased from two to seven. This color change allowed for easy visual assessment of the melon’s pH level and freshness.

In drupe, Maftoonazad and Ramaswamy used a natural pigment derived from red cabbage to act as a pH biosensor for monitoring the freshness of date *Rutab* [147]. The intelligent packaging they formed incorporated this natural pH biosensor, enabling the detection of pH-related changes over time.

Notably, although the chemical diversity of natural pH indicators in these intelligent edible gel-based packaging offers versatile solutions for different fruit types, challenges such as environmental stability (e.g., light, temperature, humidity) and matrix compatibility persist. Regarding temperature and humidity fluctuations, rapid temperature changes damage the gel’s 3D network structure, causing indicator leakage, while high humidity weakens bonding, leading to color fading [73]. Therefore, enhancing system stability relies on strengthening interactions between indicators and biopolymer matrices, which governs their dispersion, migration, and long-term response reliability. For example, designing electrostatic interactions and multi-hydrogen-bond networks can effectively anchor indicator molecules, mitigate hydrolysis and diffusion while improving film hydrophobicity [31]. In addition, optimized pore size is also required to avoid overly large pores causing rapid indicator loss and excessively small pores hindering proton transfer and color response.

**Table 4:** Applications of intelligent edible gel-based packaging integrated with natural pH indicators for fruit quality monitoring and preservation.

Fruit Category	Fruit Type		Natural pH Indicator (Source)	Intelligent Packaging Matrices	Results		References
					Preservation Effect	pH-Sensitive Behavior	
Fresh whole fruit	Drupe	Litchis	Anthocyanins (blueberry extract)	Cellulose nanofibers, Sodium alginate and κ-carrageenan	Prolonged the shelf life by 6 days (25 °C)	Purple to pink (as freshness declines)	[148]
	Berries and drupe	Red grapes and plums	Anthocyanin (pomegranate extract)	Carboxymethyl cellulose (CMC)	Prolonged the shelf lives of red grapes and plums by up to 25 days (4 °C)	Red (pH 2) to caramel-colored (pH 6–8) to light brown (pH 10) to moss green (pH 12)	[145]
	Berries	Blueberries	Anthocyanins (blueberry extract)	Pectin-sodium and alginate-xanthan gum	Prolonged the storage life to over 18 days	Purple to light pink (as the storage pH shifted from neutral to acidic)	[128]
	Berries	Tomato	Anthocyanins (mangosteen peel extract)	Polyvinyl alcohol (PVA) and glycerol	N/A	Yellow to reddish-orange (as freshness decline)	[13]
	Berries	Cherry tomatoes	Anthocyanins (blueberry extract)	CMC	Cherry tomatoes maintained weight and firmness during a 15-day storage	Film transparent to dark brown (pH 1 to pH 13).	[149]
	Berries	Banana	Betalains (red dragon fruit peel extract)	Chitosan	Enhanced film mechanical and barrier properties, delayed fruit ripening and senescence	Red (pH 1–12) to yellow (pH 13)	[150]
Fresh-cut fruit	Drupe	Fresh-cut apple pieces	Anthocyanins (red cabbage, sweet potato and butterfly pea extract)	Chitosan, lactic acid, κ-carrageenan and sweet potato extraction	Enhanced film mechanical properties	Red (pH < 3) to purple (pH 6) to dark purple (pH 7) to yellow (pH > 7)	[129]
	Berries	Durian pulp	Anthocyanins (red cabbage and butterfly pea extract)	ι-carrageenan	N/A	Deep purple (day 4) to red (day 6, pH < 4)	[151]

## 6. Conclusions

Intelligent edible gel-based packaging integrated with natural pH-sensitive indicators (e.g., anthocyanins, betanin, curcumin) is a novel food packaging approach. By enabling real-time visual freshness monitoring via reversible pH-sensitive color changes and forming a bioactive barrier on food surfaces, this technology effectively extends shelf life while maintaining product quality. Derived from abundant plant sources, natural pH-sensitive pigments offer dual advantages of intuitive quality assessment and enhanced food safety.

However, the translation of this promising technology from laboratory prototypes to mainstream commercial applications is contingent upon overcoming a series of interconnected scientific, engineering, and market challenges. Beyond the intrinsic material limitations commonly cited—such as insufficient mechanical strength and water vapor barrier properties—the path to industrial adoption presents more profound hurdles. Firstly, scaling up from batch-processing methods (e.g., solvent casting) to cost-effective, high-speed, and continuous manufacturing processes (e.g., extrusion-coating) is a critical engineering challenge. This necessitates ensuring the uniform dispersion of sensitive pigments and maintaining consistent, reliable pH-response performance across large production batches. Secondly, navigating the complex regulatory landscape is paramount. As the color change is directly linked to food quality perception, comprehensive safety assessments under FDA requirements are required to validate the migration safety of the indicators, their degradation products, and the scientific correlation between color change and specific food spoilage states. Finally, consumer acceptance is the ultimate gatekeeper for success. Clear communication of the indicator’s meaning and ensuring the packaging is perceived as safe, natural, and non-intrusive to the sensory quality of the food are essential to build trust and drive market adoption.

To bridge this gap, future research must adopt a holistic and application-oriented paradigm. Material innovation should focus on designing gel-based packaging that balances functionality (indication and barrier) with processability. Addressing material limitations through cross-disciplinary innovation will help turn lab-scale prototypes into commercial products.

In summary, pH-sensitive intelligent edible gel-based packaging holds immense promise for reducing food waste through proactive freshness management. By addressing material limitations via innovative composite designs and scalable processing, this technology can transition from experimental prototypes to mainstream applications, aligning with global demands for sustainable, consumer-centric food solutions.

## Figures and Tables

**Table 1:** Key properties essential for natural edible gel-based packaging matrices.

Matrices Category	Type	Oxygen Permeability (cm^3^·mm/m^3^day•kPa)	Minimum Tensile Strength (MPa)	Water Contact Angle (°)	Gelation Ability	References
Polysaccharide	Starch	15–30	2.5–5.0	30–45	High	[16,17]
	Chitosan	5–12	4.0–8.0	55–70	High	[18,19]
Protein-base	Gelatin	3–8	6.0–10.0	45–60	High	[20,21]
	Soybean protein	10–15	2.0–4.0	35–50	Medium	[22,23]
Lipid-base	beeswax	0.5–2.0	0.5–1.5	85–95	Low	[24,25]
Composite	Chitosan-beeswax composite	2–5	5.0–9.0	70–80	Medium to high	[26,27]
	Pectin-carrageenan composite	4–9	3.5–7.0	50–65	High	[28]

**Table 3:** Comparison of molding techniques for edible films/coatings.

Molding Technique	Application Scenarios for Edible Films/Coatings	Advantages and Disadvantages	Preparation Methods for Synthetic Films (e.g., PE/PP/PET)	Core Differences	References
Electrospinning	Nanofibrous gel films	Adv.: High specific surface area, high pigment loading capacity, enhanced gas barrier properties Disadv.: Low production efficiency, high equipment cost, limited scalability for mass production	Melt spinning	Edible films/coatings use natural biopolymers, while synthetic films rely on petroleum-derived polymers; edible films use low-temperature aqueous/alcoholic solvents, while synthetic films use high-temperature organic solvents	[127,128]
3D Printing	Customized edible gel films	Adv.: Personalized design, almost no material waste, precise control of film thickness Disadv.: Slow printing speed, need for specialized gel inks	Fused deposition modeling (FDM)	Edible gel inks are biodegradable and food-compatible, while synthetic film inks contain non-degradable plastic particles and are non-edible	[129,130]
Continuous casting	Pilot-scale production of edible gel films	Adv.: Higher efficiency than batch casting, uniform film thickness, suitable for industrial scaling Disadv.: Requires precise control of conveyor belt speed and drying temperature	Extrusion molding	Edible films are prepared at low temperatures (protecting natural pH indicator pigments), while synthetic film extrusion use high temperatures; edible films use water-based solvents, while extrusion molding use fossil fuel-derived solvents	[103,131]
Compression molding	High-mechanical-strength films (e.g., starch-gelatin composite gels)	Adv.: Enhanced mechanical robustness; improved water resistance Disadv.: Unsuitable for heat-sensitive pigments (e.g., anthocyanins) and requires high-pressure molds	Hot press molding	Edible films maintain biocompatibility at high temperatures, while synthetic films release toxic byproducts during hot pressing; edible films are biodegradable post-use	[132,133]

PE: Polyethylene; PP: Polypropylene; PET: Polyethylene terephthalate.

## Data Availability

The original contributions presented in this study are included in the article. Further inquiries can be directed to the corresponding author.
